# Editorial: Next generation *in vitro* models of the human blood - brain/cerebrospinal fluid barrier

**DOI:** 10.3389/fnmol.2024.1371733

**Published:** 2024-01-30

**Authors:** Randy S. Daughters, Lisa Julian, Erin Knock, Stephanie M. Willerth

**Affiliations:** ^1^Emulate Bio, Inc., Boston, MA, United States; ^2^Stem Cell Institute, University of Minnesota Medical School, Minneapolis, MN, United States; ^3^Department of Biological Sciences, Simon Fraser University, Burnaby, BC, Canada; ^4^Institute for Neuroscience and Neurotechnology, Simon Fraser University, Burnaby, BC, Canada; ^5^Centre for Cell Biology, Development and Disease, Simon Fraser University, Burnaby, BC, Canada; ^6^STEMCELL Technologies, Vancouver, BC, Canada; ^7^Department of Mechanical Engineering and Division of Medical Sciences, University of Victoria, Victoria, BC, Canada; ^8^School of Biomedical Engineering, University of British Columbia, Vancouver, BC, Canada

**Keywords:** neuroscience, drug discovery, lab-on-a-chip, stem cells, neurodegenerative diseases

The brain and spinal cord compose the central nervous system (CNS). The CNS plays a crucial role in coordinating function in the human body and the blood-brain barrier (BBB) provides a protective layer to ensure these organs maintain their integrity. The BBB serves as a selective barrier to prevent toxins and other harmful agents from reaching these organs while still permitting access to the nutrients that are necessary to keep these tissues alive. This protective role, however, makes it difficult to deliver therapeutic agents to the brain and spinal cord. The BBB is a heterogenous structure containing a variety of cell types–including endothelial cells, immune cells, and astrocytes–that each have unique biological roles and work together to maintain proper BBB function (Daneman and Prat, 2015). Additionally, the presence of cerebrospinal fluid (CSF) helps maintain CNS integrity, and its relationship with blood and the BBB remains dynamic. Given its complexity and critical function, disruption of the BBB can have serious implications on the health of the brain along with the rest of the body. Fittingly, a role for BBB dysfunction in multiple neurodegenerative diseases was recently reviewed (Yuan et al., 2023). One challenge is how to model these structures in an *in vitro* setting to permit detailed analysis of the relationship between CNS health and the BBB. The following collection of papers provides an interesting set of perspectives on how to study different aspects of BBB biology by highlighting advances in rodent models, human pluripotent stem cell (hPSC) differentiation, organoid and bioengineering technologies.

Nair et al. developed a human BBB-on-a-chip model as a tool to study neuroinflammation in a 3D system with perfusion–providing significant advantages over traditional 2D transwell systems. They used the Organoplates from MIMETAS to produce vessels that mimic the BBB by adding primary human brain microvascular endothelial cells (hBMECs) into these devices and allowing them to form lumenized microvessels in a highly parallel fashion. They then treated these microtissues with pro-inflammatory cytokines to induce inflammation and characterized the resulting changes to microvessel properties. They observed altered morphology of the hBMECs along with a decrease in trans-endothelial electrical resistance (TEER) and an increase in permeability. When monocytes were infused into these BBB models under inflammatory conditions they could detect enhanced adhesion to the hBMECs. Additionally, more T cells were found to migrate across these BBB models under inflammatory conditions and this effect could be reversed through treatment with a compound (Natalizumab) that blocks the VLA-4 receptor to prevent T cell binding to the hBMECs. This system demonstrates how fluid flow plays an important role in maintaining BBB properties and provides a versatile 3D system for studying the healthy and inflamed BBB. Notably, other cell types like neurons and astrocytes can be included to more closely model the *in vivo* BBB. The highly parallelized nature of the model also lends itself to applications in drug discovery.

In related work, Chew et al. provide a set of detailed methods for extracting and analyzing fluid obtained from choroid plexus organoids derived from human pluripotent stem cells. They also detail the process for generating these choroid plexus organoids from human induced pluripotent stem cells (hiPSCs). These organoids contain a cyst-like structure that generates fluid with properties similar to CSF. They provide two different strategies to extract this fluid—(1) a spin column method compatible with smaller organoids and (2) a syringe extraction method for larger organoids. They also show that the CSF-like fluid expresses appropriate markers as indicated by western blotting and mass spectrometry. This study highlights how 3D organoid systems can be used as a tool to study the blood-CSF barrier, providing further insight into its role in the healthy and diseased nervous system.

Hernández-Parra et al. provide a mini-review examining how COVID-19 alters the function of the BBB and the implications for drug delivery into the brain. Their goal is to identify how SARS-COV2 can infect hBMECs and the effects of infection on the integrity of the BBB and drug permeability into the CNS. The literature suggests that infection results in secretion of matrix metalloproteinase-9 (MMP-9), which in turn degrades type IV collagen—resulting in a loss of integrity of the BBB. This breakdown leads to systemic inflammation caused by a “cytokine storm” of proinflammatory molecules released by the peripheral immune system. These effects have significant implications for drug dosing and for drugs that normally do not cross the BBB. The authors recommend that health care professionals give thought to potential harms caused by standard treatments that might erroneously cross into the brain given the strong evidence of BBB disruption following a COVID-19 infection.

Miyata from Kyoto University provides a review of how glia cells function in the context of blood-brain communication at the circumventricular organs. The circumventricular organs located around the ventricles of the brain provide a direct interface between the brain and the blood, as they lack the BBB. These organs play both sensory and secretory roles in the CNS, helping to maintain homeostasis while still preventing the free flow of materials from the blood into the CSF. A combination of astrocytes and tanycytes (a type of radial glia) create dense walls that do not allow passage of high molecular weight (>10,000 Da) compounds from the adjacent capillaries. This review focuses on the role of macrophages and microglia in enabling communication via cell signaling between these regions and the rest of the body in both healthy and inflammatory states. The review highlights the importance of modeling the cell-cell interactions that occur in this region of the brain.

Perez-Ternero et al. produced a novel mouse model with global deletion of the C-type natriuretic peptide (CNP) as a way to determine its function in the context of the BBB in both young and aged mice. CNP regulates blood flow, but its role in the nervous system is not well understood. When CNP expression was knocked out in young mice, they experienced weight loss along with poor balance and hyperactivity. Older mice lacking CNP had seizures along with abnormal gait and sensory defects. Both sets of animals exhibited increases in permeability of the BBB compared to wild type animals and this increase could be reversed with administration of soluble CNP. *In vitro* experiments showed abnormal ZO1 expression, suggesting impaired tight junction function. This study illustrates the importance of systemic factors like CNP in maintaining BBB integrity and further studies can build upon this work to characterize its role in the CNS.

In a research study, Guo et al. investigate the mechanism by which dexmedetomidine (DEX), an adrenergic a2 receptor agonist, provides protection after intracerebral hemorrhage (ICH) in the context of a rat model. This study found DEX ameliorated behavioral and motor phenotypes found after ICH as well as a reduction in apoptosis induced neuron loss. DEX treatment reduced the number of IBA1+ cells in the brain compared to control animals. Additionally, the treated microglia expressed lower iNOS and higher CD206 levels, indicating a potential M2 phenotype. DEX treatment also resulted in lower levels of proinflammatory cytokines. This paper represents the first study to link the neuroprotective effects of DEX to an increase in BBB integrity through increased M2 microglia activation and the NfKb pathway, providing important insight into its neuroprotective mechanism.

The final paper of this collection comes from Presset et al. who provide the first metabolomic signature of blood brain barrier opening induced by the novel technology microbubble-assisted ultrasound. Microbubble assisted ultrasound can transiently and precisely open the BBB in a specific location. This study aimed to understand the systemic metabolic effects of a localized BBB disruption in rats using state of the art proteomics analysis. In particular, they examined the metabolomics of the CSF, blood serum, and urine that were collected during sacrifice of rats in three experimental groups at 3 h, 2 days, and 1 week after BBB opening and compared to a control group where no ultrasound was applied. They were able to identify metabolites unique to the striatum, blood, urine and CSF. Blood and CSF had the highest proportion of common metabolites, as expected. The CSF metabolome returned to baseline levels, quickly followed by blood with the urine metabolome remaining disrupted for 1 week post treatment. Further work will examine the long-term effects of this type of treatment. This study provides an important workflow for determining metabolomic changes due to disruptions in the BBB–whether it is induced by external treatment or due to disease states.

Overall, this collection of articles presents multiple technological advances that highlight the potential for development of physiologically relevant models of the BBB, which can provide important biological insights and serve as preclinical tools to assess the efficacy of new potential treatments. Such models are becoming increasingly important as legislation is passed to enable the testing of drugs while reducing the use of animal models. A primary goal of next generation BBB models should be to improve relevance to human physiology in order to ensure only high-quality drug targets move forward for evaluation in clinical trials.

## Author contributions

RD: Conceptualization, Writing – review & editing. LJ: Conceptualization, Writing – review & editing. EK: Conceptualization, Writing – original draft, Writing – review & editing. SW: Conceptualization, Writing – original draft, Writing – review & editing.
